# (4-Chloro­benzoato)tris­(2-methyl-2-phenyl­prop­yl)tin(IV)

**DOI:** 10.1107/S160053681005107X

**Published:** 2010-12-15

**Authors:** Min Yang

**Affiliations:** a Department of Chemistry, Dezhou University, Dezhou Shandong 253023, People’s Republic of China

## Abstract

The title compound, [Sn(C_10_H_13_)_3_(C_7_H_4_ClO_2_)], crystallized with two independent mol­ecules per asymmetric unit. In each mol­ecule, the Sn^IV^ atom is four-coordinate and possesses a distorted tetra­hedral geometry. One of the phenyl rings of one molecule is equally disordered over two positions.

## Related literature

For tris­(2-methyl-2-phenyl­prop­yl)tin aryl­carboxyl­ates, see: Fang *et al.* (2001 ▶); Bomfim *et al.* (2002 ▶); Tian *et al.* (2005 ▶); Dong (2008 ▶).
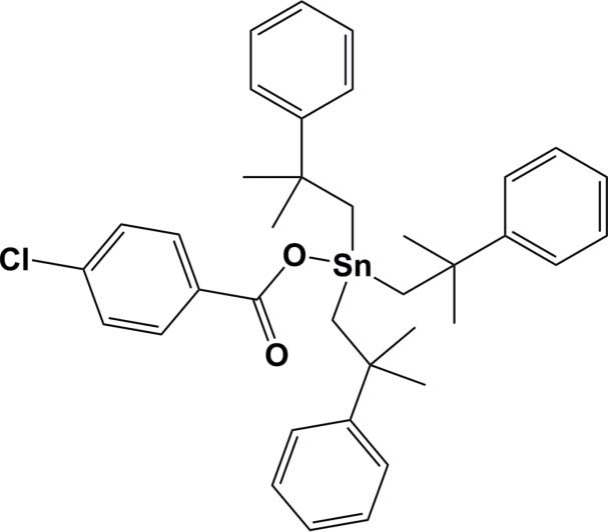


## Experimental

### 

#### Crystal data

[Sn(C_10_H_13_)_3_(C_7_H_4_ClO_2_)]
*M* *_r_* = 673.85
Triclinic, 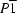

*a* = 9.948 (2) Å
*b* = 18.228 (3) Å
*c* = 19.039 (3) Å
α = 84.737 (2)°
β = 82.862 (2)°
γ = 86.127 (2)°
*V* = 3405.7 (10) Å^3^
*Z* = 4
Mo *K*α radiation
μ = 0.86 mm^−1^
*T* = 296 K
0.49 × 0.38 × 0.27 mm

#### Data collection

Bruker APEX CCD area-detector diffractometer
Absorption correction: multi-scan (*SADABS*; Sheldrick, 1996 ▶) *T*_min_ = 0.677, *T*_max_ = 0.801
21577 measured reflections
15434 independent reflections
12635 reflections with *I* > 2σ(*I*)
*R*_int_ = 0.014

#### Refinement

*R*[*F* ^2^ > 2σ(*F* ^2^)] = 0.027
*wR*(*F* ^2^) = 0.072
*S* = 1.01
15434 reflections
770 parameters
H-atom parameters constrained
Δρ_max_ = 0.41 e Å^−3^
Δρ_min_ = −0.32 e Å^−3^

### 

Data collection: *SMART* (Bruker, 2007 ▶); cell refinement: *SAINT* (Bruker, 2007 ▶); data reduction: *SAINT*; program(s) used to solve structure: *SIR97* (Altomare *et al.*, 1999 ▶); program(s) used to refine structure: *SHELXL97* (Sheldrick, 2008 ▶); molecular graphics: *SHELXTL* (Sheldrick, 2008 ▶); software used to prepare material for publication: *WinGX* (Farrugia, 1999 ▶).

## Supplementary Material

Crystal structure: contains datablocks global, I. DOI: 10.1107/S160053681005107X/su2232sup1.cif

Structure factors: contains datablocks I. DOI: 10.1107/S160053681005107X/su2232Isup2.hkl

Additional supplementary materials:  crystallographic information; 3D view; checkCIF report

## Figures and Tables

**Fig. 1.**
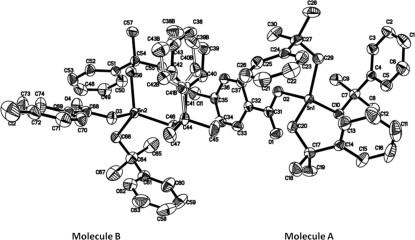
A view of the molecular structure of the two independent molecules (A and B) of the title compound, showing the atom-numbering scheme. Displacement ellipsoids are drawn at the 30% probability level. H atoms have been omitted for clarity.

## supplementary crystallographic information

### Comment

Several structures of tris(2-methyl-2-phenylpropyl)tin carboxylates have been reported, such as tris(2-methyl-2-phenylpropyl)tin arylcarboxylate with an organogermyl substituent (Fang *et al.*, 2001), acetate (Bomfim *et al.*, 2002), pyridine-3-carboxylate (Tian *et al.*, 2005) and 4-Formyl-2-methoxyphenolato (Dong, 2008). The tin(IV) atoms in these complexes all possess tetrahedral geometry.

In the two independent molecules (A and B) of the title compound, each tin(IV) atom also has a distorted tetrahedral coordination sphere (Fig. 1).

In the title compound, the C–Sn–C angles vary from 112.11 (8)° to 117.51 (8)° in molecule A and from 114.20 (9)° to 119.33 (8)° in molecule B. The O–Sn–C angle ranges from 100.11 (8)° to 104.55 (7)° in molecule A and 98.90 (7)°to 104.34 (8)° in molecule B.

The three Sn–C distances are almost equal [from 2.149 (2) to 2.153 (2) Å in molecule A and 2.150 (2) - 2.161 (2) Å in molecule B]. The Sn–O bond distances [2.0869 (14) and 2.0711 (15) Å in A and B, respectively] are similar to the values found in the carboxylate structures mentioned above.

### Experimental

The title compound was synthesized by the reaction of bis[tri(2-phenyl- 2-methylpropyl)tin]oxide (1.05 g, 1 mmol) and 4-chlorobenzoic acid (0.16 g, 1 mmol) in benzene (50 ml). Water was removed with a Dean-Stark trap.The condensation was complete in about 4 h. The resulting clear solution was evaporated under reduced pressure. The white solid obtained was purified by recrystallization from ethanol. The crystals of title compound were obtained from dichloromethane-ethanol(V/V, 1:1) by slow evaporation at 298 K.

### Refinement

One of the phenyl rings (C38-C43) in Molecule B is positionally disordered; occupancies 0.50 (3) / 0.50 (3). Like phenyl ring (C69-C74) they were refined as idealized hexagons. The hydrogen atoms were placed at calculated positions (C–H = 0.93, 0.96 and 0.97 Å, for CH, CH_3_ and CH_2_ H-atoms, respectively) and refined as riding with *U*_iso_(H) = k × *U*_eq_(C), where k = 1.5 for CH_3_ H-atoms, and = 1.2 for all other H-atoms.

### Crystal data

**Table:**

[Sn(C_10_H_13_)_3_(C_7_H_4_ClO_2_)]	*V* = 3405.7 (10) Å^3^
*M**_r_* = 673.85	*Z* = 4
Triclinic, *P*1	*F*(000) = 1392
*a* = 9.948 (2) Å	*D*_x_ = 1.314 Mg m^−^^3^
*b* = 18.228 (3) Å	Mo *K*α radiation, λ = 0.71069 Å
*c* = 19.039 (3) Å	µ = 0.86 mm^−^^1^
α = 84.737 (2)°	*T* = 296 K
β = 82.862 (2)°	Block, colorless
γ = 86.127 (2)°	0.49 × 0.38 × 0.27 mm

### Data collection

**Table:**

Bruker APEX CCD area-detector diffractometer	15434 independent reflections
Radiation source: fine-focus sealed tube	12635 reflections with *I* > 2σ(*I*)
graphite	*R*_int_ = 0.014
φ and ω scans	θ_max_ = 28.8°, θ_min_ = 1.6°
Absorption correction: multi-scan (*SADABS*; Sheldrick, 1996))	*h* = −13→13
*T*_min_ = 0.677, *T*_max_ = 0.801	*k* = −20→24
21577 measured reflections	*l* = −18→25

### Refinement

**Table:**

Refinement on *F*^2^	Primary atom site location: structure-invariant direct methods
Least-squares matrix: full	Secondary atom site location: difference Fourier map
*R*[*F*^2^ > 2σ(*F*^2^)] = 0.027	Hydrogen site location: inferred from neighbouring sites
*wR*(*F*^2^) = 0.072	H-atom parameters constrained
*S* = 1.01	*w* = 1/[σ^2^(*F*_o_^2^) + (0.0332*P*)^2^ + 0.9695*P*] where *P* = (*F*_o_^2^ + 2*F*_c_^2^)/3
15434 reflections	(Δ/σ)_max_ = 0.004
770 parameters	Δρ_max_ = 0.41 e Å^−^^3^
0 restraints	Δρ_min_ = −0.32 e Å^−^^3^

### Special details

**Table:**

Geometry. All esds (except the esd in the dihedral angle between two l.s. planes) are estimated using the full covariance matrix. The cell esds are taken into account individually in the estimation of esds in distances, angles and torsion angles; correlations between esds in cell parameters are only used when they are defined by crystal symmetry. An approximate (isotropic) treatment of cell esds is used for estimating esds involving l.s. planes.
Refinement. Refinement of F^2^ against ALL reflections. The weighted R-factor wR and goodness of fit S are based on F^2^, conventional R-factors R are based on F, with F set to zero for negative F^2^. The threshold expression of F^2^ > 2sigma(F^2^) is used only for calculating R-factors(gt) etc. and is not relevant to the choice of reflections for refinement. R-factors based on F^2^ are statistically about twice as large as those based on F, and R- factors based on ALL data will be even larger.

### Fractional atomic coordinates and isotropic or equivalent isotropic displacement parameters (Å^2^)

**Table:**

	*x*	*y*	*z*	*U*_iso_*/*U*_eq_	Occ. (<1)
Sn1	0.604277 (13)	0.315748 (7)	0.287103 (7)	0.04180 (4)	
Cl1	0.73721 (8)	0.62779 (4)	−0.08746 (3)	0.07544 (18)	
O1	0.46507 (17)	0.44265 (10)	0.21057 (9)	0.0707 (5)	
O2	0.66981 (15)	0.38564 (8)	0.19820 (8)	0.0548 (4)	
C1	0.7017 (3)	0.01782 (15)	0.32657 (16)	0.0816 (8)	
H1	0.7471	−0.0196	0.3525	0.098*	
C2	0.7492 (3)	0.03787 (14)	0.25798 (16)	0.0737 (7)	
H2	0.8277	0.0141	0.2371	0.088*	
C3	0.6821 (2)	0.09319 (13)	0.21890 (13)	0.0586 (6)	
H3	0.7157	0.1057	0.1718	0.070*	
C4	0.5656 (2)	0.13042 (11)	0.24853 (11)	0.0451 (4)	
C5	0.5196 (3)	0.10867 (13)	0.31887 (12)	0.0600 (6)	
H5	0.4416	0.1322	0.3406	0.072*	
C6	0.5870 (3)	0.05312 (15)	0.35704 (14)	0.0762 (8)	
H6	0.5539	0.0396	0.4040	0.091*	
C7	0.4892 (2)	0.19172 (12)	0.20678 (11)	0.0483 (5)	
C8	0.3590 (3)	0.16053 (15)	0.18770 (15)	0.0699 (7)	
H8A	0.3102	0.1981	0.1606	0.105*	
H8B	0.3029	0.1451	0.2305	0.105*	
H8C	0.3830	0.1191	0.1601	0.105*	
C9	0.5728 (3)	0.21961 (15)	0.13734 (12)	0.0652 (6)	
H9A	0.5225	0.2594	0.1140	0.098*	
H9B	0.5915	0.1801	0.1069	0.098*	
H9C	0.6567	0.2368	0.1478	0.098*	
C10	0.4468 (2)	0.25806 (11)	0.25148 (11)	0.0468 (5)	
H10A	0.3874	0.2406	0.2931	0.056*	
H10B	0.3934	0.2936	0.2237	0.056*	
C11	0.3907 (6)	0.1524 (2)	0.51005 (19)	0.1123 (15)	
H11	0.3868	0.1043	0.5309	0.135*	
C12	0.5056 (4)	0.18836 (19)	0.50590 (18)	0.1009 (11)	
H12	0.5816	0.1650	0.5239	0.121*	
C13	0.5110 (3)	0.26048 (15)	0.47461 (15)	0.0729 (7)	
H13	0.5911	0.2846	0.4722	0.088*	
C14	0.4011 (2)	0.29679 (12)	0.44727 (11)	0.0484 (5)	
C15	0.2850 (3)	0.25851 (17)	0.45227 (15)	0.0771 (8)	
H15	0.2081	0.2809	0.4344	0.092*	
C16	0.2822 (5)	0.1866 (2)	0.4839 (2)	0.1098 (13)	
H16	0.2028	0.1617	0.4869	0.132*	
C17	0.4086 (2)	0.37643 (12)	0.41580 (12)	0.0509 (5)	
C18	0.3972 (3)	0.42612 (14)	0.47749 (15)	0.0792 (8)	
H18A	0.4038	0.4767	0.4589	0.119*	
H18B	0.4692	0.4123	0.5061	0.119*	
H18C	0.3113	0.4204	0.5060	0.119*	
C19	0.2935 (3)	0.40007 (17)	0.37064 (16)	0.0782 (8)	
H19A	0.3066	0.4490	0.3486	0.117*	
H19B	0.2082	0.3996	0.4004	0.117*	
H19C	0.2935	0.3664	0.3347	0.117*	
C20	0.5458 (2)	0.38822 (12)	0.36990 (12)	0.0515 (5)	
H20A	0.6162	0.3836	0.4013	0.062*	
H20B	0.5436	0.4385	0.3482	0.062*	
C21	0.8805 (3)	0.3999 (2)	0.4923 (2)	0.0913 (10)	
H21	0.8717	0.4261	0.5325	0.110*	
C22	0.8680 (3)	0.3258 (2)	0.49907 (16)	0.0895 (9)	
H22	0.8509	0.3011	0.5439	0.107*	
C23	0.8808 (3)	0.28671 (15)	0.43886 (14)	0.0681 (7)	
H23	0.8707	0.2361	0.4442	0.082*	
C24	0.9080 (2)	0.32119 (13)	0.37143 (12)	0.0508 (5)	
C25	0.9195 (2)	0.39718 (14)	0.36699 (15)	0.0649 (6)	
H25	0.9369	0.4228	0.3226	0.078*	
C26	0.9056 (3)	0.43545 (17)	0.4271 (2)	0.0846 (9)	
H26	0.9136	0.4862	0.4226	0.102*	
C27	0.9225 (2)	0.27742 (13)	0.30628 (13)	0.0565 (5)	
C28	1.0252 (3)	0.21041 (18)	0.31650 (19)	0.0896 (10)	
H28A	1.0357	0.1832	0.2751	0.134*	
H28B	0.9921	0.1789	0.3573	0.134*	
H28C	1.1113	0.2276	0.3234	0.134*	
C29	0.7861 (2)	0.24628 (12)	0.29662 (13)	0.0537 (5)	
H29A	0.7653	0.2102	0.3365	0.064*	
H29B	0.8021	0.2193	0.2545	0.064*	
C30	0.9746 (3)	0.32333 (19)	0.23819 (15)	0.0869 (9)	
H30A	0.9818	0.2935	0.1987	0.130*	
H30B	1.0621	0.3402	0.2428	0.130*	
H30C	0.9124	0.3650	0.2303	0.130*	
C31	0.5783 (2)	0.43568 (12)	0.17908 (12)	0.0509 (5)	
C32	0.6222 (2)	0.48453 (11)	0.11350 (11)	0.0455 (4)	
C33	0.5337 (2)	0.54120 (13)	0.09034 (13)	0.0627 (6)	
H33	0.4493	0.5492	0.1164	0.075*	
C34	0.5693 (3)	0.58594 (13)	0.02913 (14)	0.0645 (6)	
H34	0.5100	0.6242	0.0142	0.077*	
C35	0.6932 (2)	0.57295 (12)	−0.00921 (11)	0.0518 (5)	
C36	0.7831 (2)	0.51753 (12)	0.01252 (12)	0.0557 (5)	
H36	0.8670	0.5096	−0.0140	0.067*	
C37	0.7472 (2)	0.47360 (11)	0.07437 (11)	0.0507 (5)	
H37	0.8080	0.4363	0.0897	0.061*	
Sn2	0.864862 (13)	0.777477 (7)	0.166474 (7)	0.04119 (4)	
Cl2	1.21565 (12)	0.98019 (7)	0.49069 (6)	0.1369 (4)	
O3	0.91890 (15)	0.82223 (8)	0.25439 (8)	0.0523 (3)	
O4	1.02901 (19)	0.90537 (9)	0.18023 (9)	0.0666 (4)	
C38	1.1546 (9)	0.5307 (8)	0.2238 (9)	0.092 (5)	0.50 (3)
H38	1.2373	0.5062	0.2098	0.110*	0.50 (3)
C39	1.0408 (13)	0.5189 (5)	0.1916 (7)	0.081 (3)	0.50 (3)
H39	1.0474	0.4866	0.1562	0.097*	0.50 (3)
C40	0.9171 (10)	0.5555 (5)	0.2125 (6)	0.0628 (19)	0.50 (3)
H40	0.8409	0.5476	0.1910	0.075*	0.50 (3)
C41	0.9072 (6)	0.6038 (6)	0.2655 (7)	0.049 (2)	0.50 (3)
C42	1.0210 (9)	0.6156 (7)	0.2977 (7)	0.071 (2)	0.50 (3)
H42	1.0144	0.6480	0.3331	0.085*	0.50 (3)
C43	1.1447 (7)	0.5791 (9)	0.2768 (9)	0.094 (5)	0.50 (3)
H43	1.2208	0.5869	0.2983	0.113*	0.50 (3)
C38B	1.1937 (14)	0.5613 (10)	0.2515 (7)	0.092 (5)	0.50 (3)
H38B	1.2840	0.5437	0.2451	0.110*	0.50 (3)
C39B	1.1001 (19)	0.5360 (6)	0.2123 (6)	0.090 (5)	0.50 (3)
H39B	1.1277	0.5014	0.1797	0.108*	0.50 (3)
C40B	0.9652 (17)	0.5623 (5)	0.2220 (5)	0.068 (3)	0.50 (3)
H40B	0.9025	0.5453	0.1958	0.081*	0.50 (3)
C41B	0.9240 (10)	0.6139 (5)	0.2708 (6)	0.048 (2)	0.50 (3)
C42B	1.0176 (8)	0.6393 (8)	0.3100 (5)	0.065 (2)	0.50 (3)
H42B	0.9900	0.6739	0.3426	0.078*	0.50 (3)
C43B	1.1525 (9)	0.6130 (10)	0.3003 (6)	0.097 (3)	0.50 (3)
H43B	1.2151	0.6299	0.3266	0.117*	0.50 (3)
C44	0.7717 (2)	0.64468 (12)	0.28278 (12)	0.0544 (5)	
C45	0.6689 (3)	0.58349 (15)	0.30042 (17)	0.0876 (9)	
H45A	0.6762	0.5526	0.2618	0.131*	
H45B	0.5785	0.6057	0.3075	0.131*	
H45C	0.6884	0.5543	0.3429	0.131*	
C46	0.7348 (2)	0.69228 (13)	0.21576 (14)	0.0607 (6)	
H46A	0.6454	0.7157	0.2277	0.073*	
H46B	0.7264	0.6588	0.1799	0.073*	
C47	0.7527 (3)	0.69160 (15)	0.34550 (14)	0.0841 (9)	
H47A	0.6597	0.7098	0.3533	0.126*	
H47B	0.8101	0.7325	0.3357	0.126*	
H47C	0.7763	0.6623	0.3872	0.126*	
C48	1.0285 (3)	0.93124 (17)	−0.08335 (16)	0.0843 (9)	
H48	1.0146	0.9763	−0.1093	0.101*	
C49	0.9673 (3)	0.87071 (18)	−0.09744 (15)	0.0829 (9)	
H49	0.9128	0.8742	−0.1340	0.100*	
C50	0.9850 (3)	0.80381 (15)	−0.05796 (12)	0.0648 (6)	
H50	0.9422	0.7631	−0.0686	0.078*	
C51	1.0653 (2)	0.79638 (11)	−0.00293 (11)	0.0466 (5)	
C52	1.1298 (3)	0.85856 (12)	0.00955 (13)	0.0599 (6)	
H52	1.1862	0.8554	0.0453	0.072*	
C53	1.1111 (3)	0.92511 (14)	−0.03039 (16)	0.0761 (8)	
H53	1.1550	0.9660	−0.0212	0.091*	
C54	1.0854 (2)	0.72398 (11)	0.04284 (11)	0.0466 (5)	
C55	0.9899 (3)	0.66574 (13)	0.02947 (14)	0.0616 (6)	
H55A	1.0028	0.6224	0.0611	0.092*	
H55B	1.0095	0.6532	−0.0188	0.092*	
H55C	0.8976	0.6850	0.0376	0.092*	
C56	1.0625 (2)	0.73692 (12)	0.12250 (11)	0.0477 (5)	
H56A	1.1275	0.7715	0.1310	0.057*	
H56B	1.0843	0.6906	0.1491	0.057*	
C57	1.2327 (2)	0.69289 (14)	0.02523 (14)	0.0640 (6)	
H57A	1.2944	0.7281	0.0346	0.096*	
H57B	1.2484	0.6836	−0.0240	0.096*	
H57C	1.2469	0.6477	0.0541	0.096*	
C58	0.3713 (4)	0.8062 (3)	0.2973 (2)	0.1182 (14)	
H58	0.3213	0.7935	0.3408	0.142*	
C59	0.3491 (3)	0.7741 (2)	0.2395 (2)	0.1112 (13)	
H59	0.2820	0.7404	0.2429	0.133*	
C60	0.4263 (3)	0.79108 (19)	0.17427 (18)	0.0894 (9)	
H60	0.4104	0.7680	0.1347	0.107*	
C61	0.5253 (2)	0.84136 (14)	0.16742 (15)	0.0635 (6)	
C62	0.5415 (3)	0.87479 (18)	0.22809 (18)	0.0864 (9)	
H62	0.6051	0.9104	0.2252	0.104*	
C63	0.4664 (4)	0.8570 (2)	0.2926 (2)	0.1160 (13)	
H63	0.4809	0.8796	0.3326	0.139*	
C64	0.6140 (2)	0.86029 (14)	0.09763 (14)	0.0657 (7)	
C65	0.6132 (3)	0.8012 (2)	0.04466 (16)	0.0994 (11)	
H65A	0.5255	0.8027	0.0283	0.149*	
H65B	0.6329	0.7533	0.0678	0.149*	
H65C	0.6809	0.8110	0.0049	0.149*	
C66	0.7623 (2)	0.86803 (12)	0.11013 (13)	0.0562 (5)	
H66A	0.7647	0.9114	0.1358	0.067*	
H66B	0.8147	0.8776	0.0641	0.067*	
C67	0.5596 (3)	0.9349 (2)	0.0646 (2)	0.1180 (14)	
H67A	0.5629	0.9722	0.0967	0.177*	
H67B	0.4675	0.9312	0.0557	0.177*	
H67C	0.6144	0.9480	0.0206	0.177*	
C68	0.9972 (2)	0.87762 (11)	0.23994 (12)	0.0493 (5)	
C69	1.04683 (17)	0.90457 (8)	0.30302 (7)	0.0532 (5)	
C70	1.01267 (16)	0.87067 (8)	0.37072 (8)	0.0631 (6)	
H70	0.9553	0.8318	0.3773	0.076*	
C71	1.0643 (2)	0.89490 (10)	0.42856 (7)	0.0785 (8)	
H71	1.0414	0.8722	0.4739	0.094*	
C72	1.1501 (2)	0.95302 (11)	0.41871 (10)	0.0818 (9)	
C73	1.18427 (19)	0.98692 (9)	0.35101 (12)	0.0963 (10)	
H73	1.2417	1.0258	0.3444	0.116*	
C74	1.13263 (19)	0.96270 (9)	0.29316 (9)	0.0807 (8)	
H74	1.1555	0.9854	0.2479	0.097*	

### Atomic displacement parameters (Å^2^)

**Table:**

	*U*^11^	*U*^22^	*U*^33^	*U*^12^	*U*^13^	*U*^23^
Sn1	0.03670 (7)	0.04200 (8)	0.04561 (8)	−0.00403 (5)	−0.00482 (5)	0.00344 (6)
Cl1	0.0915 (5)	0.0763 (4)	0.0553 (3)	−0.0088 (3)	−0.0132 (3)	0.0210 (3)
O1	0.0563 (10)	0.0771 (11)	0.0692 (11)	0.0049 (8)	0.0091 (8)	0.0164 (9)
O2	0.0527 (8)	0.0510 (8)	0.0558 (9)	−0.0009 (7)	−0.0007 (7)	0.0117 (7)
C1	0.103 (2)	0.0613 (16)	0.0789 (19)	0.0170 (15)	−0.0220 (17)	0.0023 (14)
C2	0.0795 (18)	0.0616 (15)	0.0777 (18)	0.0185 (13)	−0.0063 (14)	−0.0135 (13)
C3	0.0654 (14)	0.0573 (13)	0.0518 (13)	0.0030 (11)	−0.0014 (11)	−0.0094 (10)
C4	0.0473 (11)	0.0444 (10)	0.0452 (11)	−0.0073 (8)	−0.0067 (9)	−0.0079 (9)
C5	0.0613 (14)	0.0635 (14)	0.0514 (13)	0.0002 (11)	0.0020 (11)	0.0009 (11)
C6	0.097 (2)	0.0717 (17)	0.0542 (14)	0.0043 (15)	−0.0057 (14)	0.0117 (13)
C7	0.0442 (11)	0.0582 (12)	0.0429 (11)	−0.0048 (9)	−0.0071 (8)	−0.0029 (9)
C8	0.0620 (15)	0.0800 (17)	0.0749 (17)	−0.0060 (13)	−0.0244 (13)	−0.0214 (14)
C9	0.0691 (15)	0.0804 (17)	0.0428 (12)	0.0039 (13)	−0.0037 (11)	0.0032 (11)
C10	0.0387 (10)	0.0514 (11)	0.0504 (11)	−0.0035 (8)	−0.0068 (9)	−0.0010 (9)
C11	0.189 (5)	0.069 (2)	0.073 (2)	−0.035 (3)	0.010 (3)	0.0125 (17)
C12	0.132 (3)	0.077 (2)	0.087 (2)	0.014 (2)	−0.014 (2)	0.0174 (18)
C13	0.0693 (16)	0.0705 (16)	0.0755 (18)	−0.0048 (13)	−0.0086 (13)	0.0132 (14)
C14	0.0466 (11)	0.0555 (12)	0.0418 (11)	−0.0082 (9)	0.0048 (9)	−0.0061 (9)
C15	0.0679 (17)	0.090 (2)	0.0731 (17)	−0.0314 (15)	0.0046 (13)	−0.0049 (15)
C16	0.130 (3)	0.103 (3)	0.095 (3)	−0.068 (3)	0.020 (2)	−0.001 (2)
C17	0.0491 (12)	0.0491 (11)	0.0525 (12)	−0.0009 (9)	0.0016 (9)	−0.0049 (9)
C18	0.103 (2)	0.0593 (15)	0.0722 (17)	−0.0045 (14)	0.0114 (15)	−0.0180 (13)
C19	0.0573 (15)	0.091 (2)	0.0809 (19)	0.0226 (14)	−0.0057 (13)	0.0009 (15)
C20	0.0537 (12)	0.0478 (11)	0.0526 (12)	−0.0121 (9)	−0.0008 (10)	−0.0018 (9)
C21	0.074 (2)	0.116 (3)	0.089 (2)	−0.0017 (19)	−0.0143 (17)	−0.039 (2)
C22	0.084 (2)	0.126 (3)	0.0592 (17)	−0.0057 (19)	−0.0140 (15)	0.0006 (18)
C23	0.0628 (15)	0.0705 (16)	0.0704 (17)	−0.0047 (12)	−0.0136 (12)	0.0058 (13)
C24	0.0325 (10)	0.0615 (13)	0.0593 (13)	−0.0035 (9)	−0.0116 (9)	−0.0008 (10)
C25	0.0554 (14)	0.0649 (15)	0.0751 (17)	−0.0092 (11)	−0.0093 (12)	−0.0037 (13)
C26	0.0687 (18)	0.0724 (18)	0.117 (3)	−0.0072 (14)	−0.0133 (18)	−0.0259 (19)
C27	0.0374 (11)	0.0688 (14)	0.0646 (14)	−0.0027 (10)	−0.0085 (10)	−0.0102 (11)
C28	0.0515 (15)	0.104 (2)	0.120 (3)	0.0240 (14)	−0.0254 (16)	−0.043 (2)
C29	0.0440 (11)	0.0519 (12)	0.0670 (14)	−0.0028 (9)	−0.0138 (10)	−0.0044 (10)
C30	0.0629 (16)	0.131 (3)	0.0669 (17)	−0.0331 (17)	0.0078 (13)	−0.0100 (17)
C31	0.0543 (13)	0.0457 (11)	0.0517 (12)	−0.0048 (9)	−0.0060 (10)	0.0018 (9)
C32	0.0506 (11)	0.0412 (10)	0.0450 (11)	−0.0030 (8)	−0.0080 (9)	−0.0013 (8)
C33	0.0549 (13)	0.0612 (14)	0.0659 (15)	0.0085 (11)	0.0015 (11)	0.0077 (11)
C34	0.0645 (15)	0.0567 (13)	0.0685 (15)	0.0103 (11)	−0.0124 (12)	0.0121 (11)
C35	0.0638 (14)	0.0476 (11)	0.0452 (11)	−0.0095 (10)	−0.0131 (10)	0.0038 (9)
C36	0.0551 (13)	0.0568 (13)	0.0525 (13)	−0.0018 (10)	−0.0025 (10)	0.0036 (10)
C37	0.0535 (12)	0.0448 (11)	0.0522 (12)	0.0032 (9)	−0.0073 (10)	0.0016 (9)
Sn2	0.04279 (8)	0.03930 (7)	0.04131 (7)	−0.00511 (5)	−0.00827 (5)	0.00398 (5)
Cl2	0.1463 (9)	0.1429 (9)	0.1476 (9)	0.0149 (7)	−0.0835 (8)	−0.0799 (8)
O3	0.0610 (9)	0.0521 (8)	0.0458 (8)	−0.0142 (7)	−0.0103 (7)	−0.0005 (6)
O4	0.0869 (12)	0.0566 (9)	0.0570 (10)	−0.0177 (8)	−0.0170 (9)	0.0134 (8)
C38	0.056 (5)	0.082 (7)	0.121 (9)	0.018 (5)	0.004 (5)	0.045 (7)
C39	0.089 (6)	0.057 (4)	0.082 (5)	0.014 (4)	0.017 (4)	0.021 (3)
C40	0.065 (4)	0.050 (3)	0.070 (4)	−0.006 (3)	−0.004 (4)	0.005 (3)
C41	0.054 (4)	0.044 (4)	0.050 (4)	−0.026 (3)	−0.010 (3)	0.021 (3)
C42	0.082 (5)	0.055 (5)	0.076 (5)	−0.011 (3)	−0.027 (4)	0.018 (4)
C43	0.061 (5)	0.098 (9)	0.117 (9)	0.002 (5)	−0.027 (5)	0.046 (8)
C38B	0.086 (6)	0.088 (7)	0.084 (6)	0.029 (7)	0.012 (5)	0.032 (5)
C39B	0.109 (10)	0.065 (6)	0.080 (5)	0.028 (6)	0.021 (6)	0.012 (4)
C40B	0.095 (7)	0.047 (3)	0.059 (4)	−0.005 (4)	0.003 (4)	−0.003 (3)
C41B	0.058 (4)	0.036 (3)	0.046 (4)	0.002 (4)	−0.002 (4)	0.012 (3)
C42B	0.068 (4)	0.064 (6)	0.062 (4)	−0.004 (3)	−0.009 (3)	0.012 (3)
C43B	0.083 (5)	0.109 (9)	0.094 (6)	0.005 (5)	−0.018 (4)	0.033 (5)
C44	0.0599 (13)	0.0444 (11)	0.0563 (13)	−0.0136 (10)	−0.0004 (10)	0.0091 (10)
C45	0.091 (2)	0.0698 (17)	0.099 (2)	−0.0373 (15)	−0.0052 (17)	0.0250 (16)
C46	0.0522 (13)	0.0543 (13)	0.0747 (16)	−0.0137 (10)	−0.0131 (11)	0.0144 (11)
C47	0.120 (2)	0.0626 (16)	0.0604 (16)	−0.0126 (16)	0.0248 (16)	−0.0009 (13)
C48	0.104 (2)	0.0670 (18)	0.0664 (18)	0.0137 (16)	0.0183 (16)	0.0239 (14)
C49	0.096 (2)	0.092 (2)	0.0553 (15)	0.0049 (17)	−0.0111 (14)	0.0174 (15)
C50	0.0762 (16)	0.0685 (15)	0.0492 (13)	−0.0085 (12)	−0.0099 (12)	0.0033 (11)
C51	0.0498 (11)	0.0455 (11)	0.0419 (10)	−0.0027 (9)	0.0035 (9)	−0.0015 (8)
C52	0.0678 (15)	0.0526 (13)	0.0575 (14)	−0.0125 (11)	0.0010 (11)	−0.0011 (11)
C53	0.099 (2)	0.0473 (13)	0.0752 (18)	−0.0141 (13)	0.0203 (16)	−0.0004 (12)
C54	0.0491 (11)	0.0420 (10)	0.0482 (11)	−0.0054 (8)	−0.0044 (9)	−0.0006 (9)
C55	0.0700 (15)	0.0491 (12)	0.0668 (15)	−0.0146 (11)	−0.0055 (12)	−0.0055 (11)
C56	0.0470 (11)	0.0488 (11)	0.0459 (11)	−0.0011 (9)	−0.0074 (9)	0.0043 (9)
C57	0.0588 (14)	0.0598 (14)	0.0694 (16)	0.0056 (11)	0.0039 (12)	−0.0052 (12)
C58	0.066 (2)	0.169 (4)	0.107 (3)	0.013 (2)	0.012 (2)	0.011 (3)
C59	0.0548 (18)	0.151 (4)	0.125 (3)	−0.026 (2)	−0.018 (2)	0.027 (3)
C60	0.0628 (17)	0.115 (3)	0.093 (2)	−0.0183 (17)	−0.0315 (16)	0.0139 (19)
C61	0.0438 (12)	0.0681 (15)	0.0785 (17)	0.0053 (11)	−0.0207 (11)	0.0067 (13)
C62	0.080 (2)	0.085 (2)	0.092 (2)	0.0026 (16)	0.0016 (17)	−0.0195 (17)
C63	0.105 (3)	0.136 (3)	0.103 (3)	0.006 (3)	0.017 (2)	−0.036 (3)
C64	0.0574 (14)	0.0727 (16)	0.0669 (15)	−0.0016 (12)	−0.0240 (12)	0.0164 (13)
C65	0.091 (2)	0.148 (3)	0.0671 (18)	−0.023 (2)	−0.0320 (17)	−0.011 (2)
C66	0.0546 (13)	0.0517 (12)	0.0609 (14)	−0.0042 (10)	−0.0144 (10)	0.0135 (10)
C67	0.081 (2)	0.121 (3)	0.143 (3)	0.0079 (19)	−0.040 (2)	0.068 (2)
C68	0.0541 (12)	0.0400 (10)	0.0541 (12)	0.0013 (9)	−0.0128 (10)	0.0007 (9)
C69	0.0560 (13)	0.0418 (11)	0.0643 (14)	0.0009 (9)	−0.0172 (11)	−0.0057 (10)
C70	0.0671 (15)	0.0653 (15)	0.0605 (14)	−0.0072 (12)	−0.0156 (12)	−0.0107 (12)
C71	0.089 (2)	0.087 (2)	0.0639 (16)	0.0057 (16)	−0.0214 (15)	−0.0193 (15)
C72	0.0799 (19)	0.0754 (18)	0.102 (2)	0.0116 (15)	−0.0426 (17)	−0.0402 (17)
C73	0.104 (2)	0.0684 (18)	0.130 (3)	−0.0220 (17)	−0.048 (2)	−0.0192 (19)
C74	0.094 (2)	0.0596 (15)	0.095 (2)	−0.0239 (14)	−0.0317 (17)	0.0033 (14)

### Geometric parameters (Å, °)

**Table:**

Sn1—O2	2.0869 (14)	O4—C68	1.214 (3)
Sn1—C20	2.149 (2)	C38—C39	1.3900
Sn1—C29	2.153 (2)	C38—C43	1.3900
Sn1—C10	2.153 (2)	C38—H38	0.9300
Cl1—C35	1.742 (2)	C39—C40	1.3900
O1—C31	1.213 (3)	C39—H39	0.9300
O2—C31	1.305 (2)	C40—C41	1.3900
C1—C2	1.358 (4)	C40—H40	0.9300
C1—C6	1.363 (4)	C41—C42	1.3900
C1—H1	0.9300	C41—C44	1.510 (6)
C2—C3	1.382 (3)	C42—C43	1.3900
C2—H2	0.9300	C42—H42	0.9300
C3—C4	1.387 (3)	C43—H43	0.9300
C3—H3	0.9300	C38B—C39B	1.3900
C4—C5	1.391 (3)	C38B—C43B	1.3900
C4—C7	1.527 (3)	C38B—H38B	0.9300
C5—C6	1.377 (3)	C39B—C40B	1.3900
C5—H5	0.9300	C39B—H39B	0.9300
C6—H6	0.9300	C40B—C41B	1.3900
C7—C9	1.536 (3)	C40B—H40B	0.9300
C7—C8	1.545 (3)	C41B—C42B	1.3900
C7—C10	1.548 (3)	C41B—C44	1.576 (8)
C8—H8A	0.9600	C42B—C43B	1.3900
C8—H8B	0.9600	C42B—H42B	0.9300
C8—H8C	0.9600	C43B—H43B	0.9300
C9—H9A	0.9600	C44—C47	1.518 (3)
C9—H9B	0.9600	C44—C46	1.545 (3)
C9—H9C	0.9600	C44—C45	1.550 (3)
C10—H10A	0.9700	C45—H45A	0.9600
C10—H10B	0.9700	C45—H45B	0.9600
C11—C16	1.337 (6)	C45—H45C	0.9600
C11—C12	1.346 (5)	C46—H46A	0.9700
C11—H11	0.9300	C46—H46B	0.9700
C12—C13	1.394 (4)	C47—H47A	0.9600
C12—H12	0.9300	C47—H47B	0.9600
C13—C14	1.372 (3)	C47—H47C	0.9600
C13—H13	0.9300	C48—C49	1.359 (4)
C14—C15	1.378 (3)	C48—C53	1.370 (4)
C14—C17	1.522 (3)	C48—H48	0.9300
C15—C16	1.392 (5)	C49—C50	1.385 (4)
C15—H15	0.9300	C49—H49	0.9300
C16—H16	0.9300	C50—C51	1.386 (3)
C17—C19	1.532 (3)	C50—H50	0.9300
C17—C18	1.536 (3)	C51—C52	1.392 (3)
C17—C20	1.543 (3)	C51—C54	1.529 (3)
C18—H18A	0.9600	C52—C53	1.386 (3)
C18—H18B	0.9600	C52—H52	0.9300
C18—H18C	0.9600	C53—H53	0.9300
C19—H19A	0.9600	C54—C55	1.530 (3)
C19—H19B	0.9600	C54—C56	1.543 (3)
C19—H19C	0.9600	C54—C57	1.543 (3)
C20—H20A	0.9700	C55—H55A	0.9600
C20—H20B	0.9700	C55—H55B	0.9600
C21—C26	1.348 (5)	C55—H55C	0.9600
C21—C22	1.358 (5)	C56—H56A	0.9700
C21—H21	0.9300	C56—H56B	0.9700
C22—C23	1.392 (4)	C57—H57A	0.9600
C22—H22	0.9300	C57—H57B	0.9600
C23—C24	1.380 (3)	C57—H57C	0.9600
C23—H23	0.9300	C58—C59	1.340 (6)
C24—C25	1.392 (3)	C58—C63	1.358 (6)
C24—C27	1.522 (3)	C58—H58	0.9300
C25—C26	1.382 (4)	C59—C60	1.398 (5)
C25—H25	0.9300	C59—H59	0.9300
C26—H26	0.9300	C60—C61	1.377 (4)
C27—C30	1.530 (4)	C60—H60	0.9300
C27—C29	1.544 (3)	C61—C62	1.386 (4)
C27—C28	1.553 (3)	C61—C64	1.526 (4)
C28—H28A	0.9600	C62—C63	1.379 (5)
C28—H28B	0.9600	C62—H62	0.9300
C28—H28C	0.9600	C63—H63	0.9300
C29—H29A	0.9700	C64—C67	1.539 (4)
C29—H29B	0.9700	C64—C66	1.541 (3)
C30—H30A	0.9600	C64—C65	1.542 (4)
C30—H30B	0.9600	C65—H65A	0.9600
C30—H30C	0.9600	C65—H65B	0.9600
C31—C32	1.502 (3)	C65—H65C	0.9600
C32—C37	1.380 (3)	C66—H66A	0.9700
C32—C33	1.388 (3)	C66—H66B	0.9700
C33—C34	1.382 (3)	C67—H67A	0.9600
C33—H33	0.9300	C67—H67B	0.9600
C34—C35	1.370 (3)	C67—H67C	0.9600
C34—H34	0.9300	C68—C69	1.489 (2)
C35—C36	1.373 (3)	C69—C70	1.3900
C36—C37	1.384 (3)	C69—C74	1.3900
C36—H36	0.9300	C70—C71	1.3900
C37—H37	0.9300	C70—H70	0.9300
Sn2—O3	2.0711 (15)	C71—C72	1.3900
Sn2—C56	2.150 (2)	C71—H71	0.9300
Sn2—C66	2.153 (2)	C72—C73	1.3900
Sn2—C46	2.161 (2)	C73—C74	1.3900
Cl2—C72	1.7144 (14)	C73—H73	0.9300
O3—C68	1.305 (2)	C74—H74	0.9300
			
O2—Sn1—C20	104.55 (7)	C40—C39—H39	120.0
O2—Sn1—C29	100.11 (8)	C38—C39—H39	120.0
C20—Sn1—C29	116.27 (9)	C39—C40—C41	120.0
O2—Sn1—C10	103.32 (7)	C39—C40—H40	120.0
C20—Sn1—C10	117.51 (8)	C41—C40—H40	120.0
C29—Sn1—C10	112.11 (8)	C40—C41—C42	120.0
C31—O2—Sn1	114.39 (13)	C40—C41—C44	117.1 (5)
C2—C1—C6	119.3 (3)	C42—C41—C44	122.8 (5)
C2—C1—H1	120.3	C43—C42—C41	120.0
C6—C1—H1	120.3	C43—C42—H42	120.0
C1—C2—C3	120.8 (2)	C41—C42—H42	120.0
C1—C2—H2	119.6	C42—C43—C38	120.0
C3—C2—H2	119.6	C42—C43—H43	120.0
C2—C3—C4	121.3 (2)	C38—C43—H43	120.0
C2—C3—H3	119.4	C39B—C38B—C43B	120.0
C4—C3—H3	119.4	C39B—C38B—H38B	120.0
C3—C4—C5	116.6 (2)	C43B—C38B—H38B	120.0
C3—C4—C7	122.52 (19)	C40B—C39B—C38B	120.0
C5—C4—C7	120.85 (19)	C40B—C39B—H39B	120.0
C6—C5—C4	121.5 (2)	C38B—C39B—H39B	120.0
C6—C5—H5	119.2	C41B—C40B—C39B	120.0
C4—C5—H5	119.2	C41B—C40B—H40B	120.0
C1—C6—C5	120.5 (3)	C39B—C40B—H40B	120.0
C1—C6—H6	119.7	C42B—C41B—C40B	120.0
C5—C6—H6	119.7	C42B—C41B—C44	118.9 (5)
C4—C7—C9	112.39 (18)	C40B—C41B—C44	121.1 (5)
C4—C7—C8	108.18 (19)	C43B—C42B—C41B	120.0
C9—C7—C8	108.05 (19)	C43B—C42B—H42B	120.0
C4—C7—C10	111.39 (17)	C41B—C42B—H42B	120.0
C9—C7—C10	108.58 (18)	C42B—C43B—C38B	120.0
C8—C7—C10	108.11 (17)	C42B—C43B—H43B	120.0
C7—C8—H8A	109.5	C38B—C43B—H43B	120.0
C7—C8—H8B	109.5	C41—C44—C47	118.6 (5)
H8A—C8—H8B	109.5	C41—C44—C46	109.1 (5)
C7—C8—H8C	109.5	C47—C44—C46	109.3 (2)
H8A—C8—H8C	109.5	C41—C44—C45	104.9 (4)
H8B—C8—H8C	109.5	C47—C44—C45	106.3 (2)
C7—C9—H9A	109.5	C46—C44—C45	108.2 (2)
C7—C9—H9B	109.5	C41—C44—C41B	10.5 (5)
H9A—C9—H9B	109.5	C47—C44—C41B	109.3 (4)
C7—C9—H9C	109.5	C46—C44—C41B	109.9 (5)
H9A—C9—H9C	109.5	C45—C44—C41B	113.6 (5)
H9B—C9—H9C	109.5	C44—C45—H45A	109.5
C7—C10—Sn1	118.18 (13)	C44—C45—H45B	109.5
C7—C10—H10A	107.8	H45A—C45—H45B	109.5
Sn1—C10—H10A	107.8	C44—C45—H45C	109.5
C7—C10—H10B	107.8	H45A—C45—H45C	109.5
Sn1—C10—H10B	107.8	H45B—C45—H45C	109.5
H10A—C10—H10B	107.1	C44—C46—Sn2	120.39 (15)
C16—C11—C12	119.4 (3)	C44—C46—H46A	107.2
C16—C11—H11	120.3	Sn2—C46—H46A	107.2
C12—C11—H11	120.3	C44—C46—H46B	107.2
C11—C12—C13	120.1 (4)	Sn2—C46—H46B	107.2
C11—C12—H12	119.9	H46A—C46—H46B	106.9
C13—C12—H12	119.9	C44—C47—H47A	109.5
C14—C13—C12	121.7 (3)	C44—C47—H47B	109.5
C14—C13—H13	119.1	H47A—C47—H47B	109.5
C12—C13—H13	119.1	C44—C47—H47C	109.5
C13—C14—C15	116.7 (2)	H47A—C47—H47C	109.5
C13—C14—C17	120.5 (2)	H47B—C47—H47C	109.5
C15—C14—C17	122.7 (2)	C49—C48—C53	119.2 (3)
C14—C15—C16	120.4 (3)	C49—C48—H48	120.4
C14—C15—H15	119.8	C53—C48—H48	120.4
C16—C15—H15	119.8	C48—C49—C50	120.8 (3)
C11—C16—C15	121.6 (3)	C48—C49—H49	119.6
C11—C16—H16	119.2	C50—C49—H49	119.6
C15—C16—H16	119.2	C49—C50—C51	121.3 (3)
C14—C17—C19	112.3 (2)	C49—C50—H50	119.4
C14—C17—C18	107.98 (19)	C51—C50—H50	119.4
C19—C17—C18	108.4 (2)	C50—C51—C52	117.0 (2)
C14—C17—C20	110.80 (17)	C50—C51—C54	122.9 (2)
C19—C17—C20	108.98 (19)	C52—C51—C54	120.0 (2)
C18—C17—C20	108.27 (19)	C53—C52—C51	121.1 (3)
C17—C18—H18A	109.5	C53—C52—H52	119.5
C17—C18—H18B	109.5	C51—C52—H52	119.5
H18A—C18—H18B	109.5	C48—C53—C52	120.6 (3)
C17—C18—H18C	109.5	C48—C53—H53	119.7
H18A—C18—H18C	109.5	C52—C53—H53	119.7
H18B—C18—H18C	109.5	C51—C54—C55	112.55 (19)
C17—C19—H19A	109.5	C51—C54—C56	110.77 (17)
C17—C19—H19B	109.5	C55—C54—C56	108.40 (17)
H19A—C19—H19B	109.5	C51—C54—C57	108.67 (17)
C17—C19—H19C	109.5	C55—C54—C57	108.13 (19)
H19A—C19—H19C	109.5	C56—C54—C57	108.20 (18)
H19B—C19—H19C	109.5	C54—C55—H55A	109.5
C17—C20—Sn1	117.25 (14)	C54—C55—H55B	109.5
C17—C20—H20A	108.0	H55A—C55—H55B	109.5
Sn1—C20—H20A	108.0	C54—C55—H55C	109.5
C17—C20—H20B	108.0	H55A—C55—H55C	109.5
Sn1—C20—H20B	108.0	H55B—C55—H55C	109.5
H20A—C20—H20B	107.2	C54—C56—Sn2	118.35 (14)
C26—C21—C22	119.8 (3)	C54—C56—H56A	107.7
C26—C21—H21	120.1	Sn2—C56—H56A	107.7
C22—C21—H21	120.1	C54—C56—H56B	107.7
C21—C22—C23	120.0 (3)	Sn2—C56—H56B	107.7
C21—C22—H22	120.0	H56A—C56—H56B	107.1
C23—C22—H22	120.0	C54—C57—H57A	109.5
C24—C23—C22	121.7 (3)	C54—C57—H57B	109.5
C24—C23—H23	119.1	H57A—C57—H57B	109.5
C22—C23—H23	119.1	C54—C57—H57C	109.5
C23—C24—C25	116.3 (2)	H57A—C57—H57C	109.5
C23—C24—C27	120.9 (2)	H57B—C57—H57C	109.5
C25—C24—C27	122.8 (2)	C59—C58—C63	120.3 (4)
C26—C25—C24	121.4 (3)	C59—C58—H58	119.8
C26—C25—H25	119.3	C63—C58—H58	119.8
C24—C25—H25	119.3	C58—C59—C60	120.3 (4)
C21—C26—C25	120.7 (3)	C58—C59—H59	119.9
C21—C26—H26	119.7	C60—C59—H59	119.9
C25—C26—H26	119.7	C61—C60—C59	121.2 (3)
C24—C27—C30	112.5 (2)	C61—C60—H60	119.4
C24—C27—C29	110.85 (18)	C59—C60—H60	119.4
C30—C27—C29	109.2 (2)	C60—C61—C62	116.3 (3)
C24—C27—C28	109.4 (2)	C60—C61—C64	123.0 (3)
C30—C27—C28	107.7 (2)	C62—C61—C64	120.7 (3)
C29—C27—C28	107.0 (2)	C63—C62—C61	122.1 (3)
C27—C28—H28A	109.5	C63—C62—H62	118.9
C27—C28—H28B	109.5	C61—C62—H62	118.9
H28A—C28—H28B	109.5	C58—C63—C62	119.6 (4)
C27—C28—H28C	109.5	C58—C63—H63	120.2
H28A—C28—H28C	109.5	C62—C63—H63	120.2
H28B—C28—H28C	109.5	C61—C64—C67	108.2 (2)
C27—C29—Sn1	122.42 (15)	C61—C64—C66	111.2 (2)
C27—C29—H29A	106.7	C67—C64—C66	108.1 (2)
Sn1—C29—H29A	106.7	C61—C64—C65	112.2 (2)
C27—C29—H29B	106.7	C67—C64—C65	109.2 (3)
Sn1—C29—H29B	106.7	C66—C64—C65	107.9 (2)
H29A—C29—H29B	106.6	C64—C65—H65A	109.5
C27—C30—H30A	109.5	C64—C65—H65B	109.5
C27—C30—H30B	109.5	H65A—C65—H65B	109.5
H30A—C30—H30B	109.5	C64—C65—H65C	109.5
C27—C30—H30C	109.5	H65A—C65—H65C	109.5
H30A—C30—H30C	109.5	H65B—C65—H65C	109.5
H30B—C30—H30C	109.5	C64—C66—Sn2	118.38 (15)
O1—C31—O2	123.5 (2)	C64—C66—H66A	107.7
O1—C31—C32	121.4 (2)	Sn2—C66—H66A	107.7
O2—C31—C32	115.01 (18)	C64—C66—H66B	107.7
C37—C32—C33	118.80 (19)	Sn2—C66—H66B	107.7
C37—C32—C31	121.85 (19)	H66A—C66—H66B	107.1
C33—C32—C31	119.33 (19)	C64—C67—H67A	109.5
C34—C33—C32	120.9 (2)	C64—C67—H67B	109.5
C34—C33—H33	119.5	H67A—C67—H67B	109.5
C32—C33—H33	119.5	C64—C67—H67C	109.5
C35—C34—C33	118.9 (2)	H67A—C67—H67C	109.5
C35—C34—H34	120.5	H67B—C67—H67C	109.5
C33—C34—H34	120.5	O4—C68—O3	123.7 (2)
C34—C35—C36	121.5 (2)	O4—C68—C69	121.73 (19)
C34—C35—Cl1	119.21 (17)	O3—C68—C69	114.57 (18)
C36—C35—Cl1	119.31 (18)	C70—C69—C74	120.0
C35—C36—C37	119.1 (2)	C70—C69—C68	120.91 (13)
C35—C36—H36	120.4	C74—C69—C68	119.04 (13)
C37—C36—H36	120.4	C71—C70—C69	120.0
C32—C37—C36	120.7 (2)	C71—C70—H70	120.0
C32—C37—H37	119.6	C69—C70—H70	120.0
C36—C37—H37	119.6	C72—C71—C70	120.0
O3—Sn2—C56	98.90 (7)	C72—C71—H71	120.0
O3—Sn2—C66	104.34 (8)	C70—C71—H71	120.0
C56—Sn2—C66	119.33 (8)	C71—C72—C73	120.0
O3—Sn2—C46	101.53 (8)	C71—C72—Cl2	118.97 (14)
C56—Sn2—C46	114.20 (9)	C73—C72—Cl2	121.01 (14)
C66—Sn2—C46	114.63 (9)	C74—C73—C72	120.0
C68—O3—Sn2	114.93 (13)	C74—C73—H73	120.0
C39—C38—C43	120.0	C72—C73—H73	120.0
C39—C38—H38	120.0	C73—C74—C69	120.0
C43—C38—H38	120.0	C73—C74—H74	120.0
C40—C39—C38	120.0	C69—C74—H74	120.0
			
C20—Sn1—O2—C31	−59.08 (17)	C39—C38—C43—C42	0.0
C29—Sn1—O2—C31	−179.79 (15)	C43B—C38B—C39B—C40B	0.0
C10—Sn1—O2—C31	64.41 (16)	C38B—C39B—C40B—C41B	0.0
C6—C1—C2—C3	−0.3 (5)	C39B—C40B—C41B—C42B	0.0
C1—C2—C3—C4	0.7 (4)	C39B—C40B—C41B—C44	−180.0 (9)
C2—C3—C4—C5	−0.7 (3)	C40B—C41B—C42B—C43B	0.0
C2—C3—C4—C7	−180.0 (2)	C44—C41B—C42B—C43B	180.0 (9)
C3—C4—C5—C6	0.2 (4)	C41B—C42B—C43B—C38B	0.0
C7—C4—C5—C6	179.6 (2)	C39B—C38B—C43B—C42B	0.0
C2—C1—C6—C5	−0.1 (5)	C40—C41—C44—C47	173.6 (4)
C4—C5—C6—C1	0.1 (4)	C42—C41—C44—C47	−9.7 (9)
C3—C4—C7—C9	−14.2 (3)	C40—C41—C44—C46	−60.5 (6)
C5—C4—C7—C9	166.5 (2)	C42—C41—C44—C46	116.2 (6)
C3—C4—C7—C8	105.0 (2)	C40—C41—C44—C45	55.2 (6)
C5—C4—C7—C8	−74.2 (3)	C42—C41—C44—C45	−128.0 (6)
C3—C4—C7—C10	−136.3 (2)	C40—C41—C44—C41B	−157 (5)
C5—C4—C7—C10	44.4 (3)	C42—C41—C44—C41B	20 (5)
C4—C7—C10—Sn1	61.6 (2)	C42B—C41B—C44—C41	−161 (5)
C9—C7—C10—Sn1	−62.7 (2)	C40B—C41B—C44—C41	19 (5)
C8—C7—C10—Sn1	−179.66 (16)	C42B—C41B—C44—C47	−7.6 (6)
O2—Sn1—C10—C7	80.87 (16)	C40B—C41B—C44—C47	172.4 (5)
C20—Sn1—C10—C7	−164.65 (14)	C42B—C41B—C44—C46	112.4 (5)
C29—Sn1—C10—C7	−26.05 (18)	C40B—C41B—C44—C46	−67.7 (7)
C16—C11—C12—C13	0.0 (6)	C42B—C41B—C44—C45	−126.2 (4)
C11—C12—C13—C14	−0.1 (5)	C40B—C41B—C44—C45	53.8 (7)
C12—C13—C14—C15	0.0 (4)	C41—C44—C46—Sn2	−60.3 (5)
C12—C13—C14—C17	177.8 (3)	C47—C44—C46—Sn2	70.8 (3)
C13—C14—C15—C16	0.1 (4)	C45—C44—C46—Sn2	−173.79 (19)
C17—C14—C15—C16	−177.7 (3)	C41B—C44—C46—Sn2	−49.2 (5)
C12—C11—C16—C15	0.1 (6)	O3—Sn2—C46—C44	−40.3 (2)
C14—C15—C16—C11	−0.1 (5)	C56—Sn2—C46—C44	65.0 (2)
C13—C14—C17—C19	164.4 (2)	C66—Sn2—C46—C44	−152.14 (18)
C15—C14—C17—C19	−18.0 (3)	C53—C48—C49—C50	1.5 (4)
C13—C14—C17—C18	−76.2 (3)	C48—C49—C50—C51	0.1 (4)
C15—C14—C17—C18	101.5 (3)	C49—C50—C51—C52	−1.6 (4)
C13—C14—C17—C20	42.2 (3)	C49—C50—C51—C54	178.8 (2)
C15—C14—C17—C20	−140.1 (2)	C50—C51—C52—C53	1.6 (3)
C14—C17—C20—Sn1	53.8 (2)	C54—C51—C52—C53	−178.8 (2)
C19—C17—C20—Sn1	−70.3 (2)	C49—C48—C53—C52	−1.5 (4)
C18—C17—C20—Sn1	172.01 (16)	C51—C52—C53—C48	−0.1 (4)
O2—Sn1—C20—C17	133.45 (16)	C50—C51—C54—C55	−9.5 (3)
C29—Sn1—C20—C17	−117.26 (17)	C52—C51—C54—C55	170.8 (2)
C10—Sn1—C20—C17	19.6 (2)	C50—C51—C54—C56	−131.1 (2)
C26—C21—C22—C23	−0.2 (5)	C52—C51—C54—C56	49.3 (3)
C21—C22—C23—C24	0.9 (4)	C50—C51—C54—C57	110.2 (2)
C22—C23—C24—C25	−1.1 (4)	C52—C51—C54—C57	−69.4 (3)
C22—C23—C24—C27	179.6 (2)	C51—C54—C56—Sn2	62.7 (2)
C23—C24—C25—C26	0.6 (3)	C55—C54—C56—Sn2	−61.3 (2)
C27—C24—C25—C26	179.9 (2)	C57—C54—C56—Sn2	−178.29 (14)
C22—C21—C26—C25	−0.3 (5)	O3—Sn2—C56—C54	−157.89 (15)
C24—C25—C26—C21	0.1 (4)	C66—Sn2—C56—C54	−45.84 (19)
C23—C24—C27—C30	−170.9 (2)	C46—Sn2—C56—C54	95.12 (16)
C25—C24—C27—C30	9.9 (3)	C63—C58—C59—C60	1.8 (6)
C23—C24—C27—C29	66.5 (3)	C58—C59—C60—C61	−0.7 (6)
C25—C24—C27—C29	−112.7 (2)	C59—C60—C61—C62	−1.3 (4)
C23—C24—C27—C28	−51.2 (3)	C59—C60—C61—C64	178.6 (3)
C25—C24—C27—C28	129.6 (2)	C60—C61—C62—C63	2.3 (5)
C24—C27—C29—Sn1	56.8 (3)	C64—C61—C62—C63	−177.6 (3)
C30—C27—C29—Sn1	−67.7 (3)	C59—C58—C63—C62	−0.8 (6)
C28—C27—C29—Sn1	175.97 (18)	C61—C62—C63—C58	−1.3 (6)
O2—Sn1—C29—C27	58.4 (2)	C60—C61—C64—C67	102.1 (3)
C20—Sn1—C29—C27	−53.5 (2)	C62—C61—C64—C67	−77.9 (3)
C10—Sn1—C29—C27	167.32 (17)	C60—C61—C64—C66	−139.3 (3)
Sn1—O2—C31—O1	1.4 (3)	C62—C61—C64—C66	40.6 (3)
Sn1—O2—C31—C32	−177.74 (14)	C60—C61—C64—C65	−18.4 (3)
O1—C31—C32—C37	−175.2 (2)	C62—C61—C64—C65	161.6 (3)
O2—C31—C32—C37	4.0 (3)	C61—C64—C66—Sn2	54.9 (3)
O1—C31—C32—C33	3.2 (3)	C67—C64—C66—Sn2	173.4 (2)
O2—C31—C32—C33	−177.7 (2)	C65—C64—C66—Sn2	−68.5 (3)
C37—C32—C33—C34	0.2 (4)	O3—Sn2—C66—C64	−120.47 (19)
C31—C32—C33—C34	−178.2 (2)	C56—Sn2—C66—C64	130.46 (19)
C32—C33—C34—C35	0.9 (4)	C46—Sn2—C66—C64	−10.3 (2)
C33—C34—C35—C36	−1.2 (4)	Sn2—O3—C68—O4	5.3 (3)
C33—C34—C35—Cl1	178.6 (2)	Sn2—O3—C68—C69	−174.14 (12)
C34—C35—C36—C37	0.4 (4)	O4—C68—C69—C70	−178.03 (17)
Cl1—C35—C36—C37	−179.38 (18)	O3—C68—C69—C70	1.5 (2)
C33—C32—C37—C36	−1.0 (3)	O4—C68—C69—C74	−0.5 (3)
C31—C32—C37—C36	177.4 (2)	O3—C68—C69—C74	179.02 (14)
C35—C36—C37—C32	0.7 (3)	C74—C69—C70—C71	0.0
C56—Sn2—O3—C68	64.39 (15)	C68—C69—C70—C71	177.53 (17)
C66—Sn2—O3—C68	−59.10 (16)	C69—C70—C71—C72	0.0
C46—Sn2—O3—C68	−178.52 (15)	C70—C71—C72—C73	0.0
C43—C38—C39—C40	0.0	C70—C71—C72—Cl2	−178.37 (15)
C38—C39—C40—C41	0.0	C71—C72—C73—C74	0.0
C39—C40—C41—C42	0.0	Cl2—C72—C73—C74	178.34 (15)
C39—C40—C41—C44	176.9 (9)	C72—C73—C74—C69	0.0
C40—C41—C42—C43	0.0	C70—C69—C74—C73	0.0
C44—C41—C42—C43	−176.7 (10)	C68—C69—C74—C73	−177.58 (16)
C41—C42—C43—C38	0.0		
